# Impacts of financial, caregiving, and emotional support on mental health: case of hypertensive patients in China

**DOI:** 10.3389/fpubh.2025.1601168

**Published:** 2025-07-24

**Authors:** Jing Tan, Deliang Kong, Lin Hu, Chuan Pu

**Affiliations:** School of Public Health, Chongqing Medical University, Chongqing, China

**Keywords:** mental health, depression, intergenerational support, older adults, hypertension

## Abstract

**Background:**

Hypertension and mental disorders, particularly depression, significantly impact Asia’s older adult. Alleviating depression in older adult hypertensive patients can significantly enhance their quality of life. This study evaluates the mental health status of hypertensive patients over 60 who do or do not provide intergenerational family support.

**Methods:**

Using 2020 CHARLS data, we analyzed 4,851 hypertensive patients aged ≥60. Intergenerational family support was categorized into financial, daily caregiving, and emotional support, with mental health measured via the Chinese CESD-10. Multivariate linear regression and propensity score matching (PSM) assessed mental health changes from different support types. Heterogeneity analysis examined differences across age, gender, education, and urban–rural areas.

**Results:**

Caregiving (*β* = −0.78, *p* < 0.01) and emotional support (*β* = −0.56, *p* < 0.05) significantly reduced depressive symptoms in older adult hypertensive patients, while financial support showed no significant impact. Sensitivity tests confirmed these results. Subgroup analyses revealed greater benefits for women (emotional support: β = −0.77), rural residents (emotional support: β = −0.59), and those aged ≥75 (caregiving support: β = −2.26).

**Conclusion:**

Non-material intergenerational support, especially caregiving and emotional involvement, is crucial for alleviating depression in older adult hypertensive patients, whereas financial support has little effect. Policies should prioritize psychosocial interventions over financial aid, particularly for vulnerable groups like women, rural residents, and the older adult aged ≥75.

## 1 Introduction

According to the World Health Organization, the number of depression and anxiety cases in the Southeast Asia region ranks first globally, accounting for 7.2 and 2.8% of all disability—adjusted life years (YLDs), respectively (1). Globally, hypertension is a leading cause of premature death and disability. High blood pressure (BP) affects 2 out of every 5 adults worldwide (2). Furthermore, several national reports from Asia indicate that hypertension, depression, and anxiety primarily occur in the older adult. The term of “triple burden” is characterized by the coexistence of being older adult, along with hypertension and mental health problems (3). Therefore, the older adult, especially in Asia, are vulnerable to the burden of hypertension and mental health issues. The “triple burden” of population aging, hypertension, and mental health problems makes the Asian older adult more vulnerable (4). In addition, older adults in Asia often have multiple chronic conditions such as cardiovascular disease and diabetes. Compared to non-diabetic patients, diabetic patients are 2–3 times more likely to develop cardiovascular disease (CVD), including coronary heart disease, cardiomyopathy, stroke, and peripheral artery disease (5, 6). Data from the Asia-Pacific Cohort Study Collaboration indicate that the association between blood pressure and cardiovascular disease is stronger in Asian patients than in white patients in Australia and New Zealand (7, 8). CVD and depression are common. Patients with CVD are more likely to develop depression than the general population (9).

There is a bidirectional relationship between depression and hypertension, leading to reduced quality of life, decreased treatment adherence, and increased mortality in older adult patients with hypertension. For the older adult, hypertension should be tackled along with depression to reduce the mortality associated with hypertension (10). A randomized study showed that hypertensive patients with depressive symptoms require more antihypertensive medications to achieve good blood pressure control (11). Contrary to the general assumption, compared with younger patients (<50 years), patients aged 65–80 have better rather than worse medication adherence. However, in very older adult patients, medication adherence tends to decrease for various reasons, one of which is the progressive cognitive decline or depression that develops with age (12). Depression itself increases the likelihood of having functional or cognitive impairments by 2–3 times (13). Moreover, the symptoms of depression are closely related to poor blood pressure control in hypertension and the development of hypertension-mediated complications (14). Research has focused on understanding the interplay between psychological health and hypertension, as well as the effectiveness of treatment interventions (15, 16). Of particular research interest is the impact of social support on mental health. So far, little attention has been paid to the mental health of hypertensive patients who do not receive intergenerational family support.

Intergenerational support, which refers to the interaction and sharing of time and emotional resources between different generations, plays an indispensable role in maintaining intergenerational relationships based on family bonds (15). Nearly 1 billion people worldwide suffer from hypertension, and this number is projected to increase to 1.5 billion by 2025 (17). Poor medication adherence and an unhealthy lifestyle are the main reasons for uncontrolled hypertension. Even among those for whom antihypertensive medications are provided free of charge, poor adherence still exists (18). Patients need lifelong treatment based on lifestyle changes and antihypertensive medications. Self-care has been recognized as an important determinant of achieving optimal blood pressure control at the individual level. Family support enhances adherence to self-care practices related to blood pressure management (19). However, more evidence is needed to understand the impact of intergenerational family support on the mental health of Asian older adult patients with hypertension.

Intergenerational family support is a type of social support, which is a comprehensive concept and can be divided into several different categories. It can be divided into family support, friend support, community support, and colleague support (20). In this paper, “older adult hypertensive patients who receive intergenerational family support” refers to hypertensive patients aged 60 and above who receive economic support, daily care, and emotional comfort from their children as forms of intergenerational family support.

A recent systematic review assessed the quantitative evidence of the association between intergenerational family support and mental health in the older adult. Although the authors reported that children’s financial support and emotional comfort were positively correlated with the mental health of the older adult, and children’s daily care was negatively correlated with the mental health of the older adult, the article emphasized that this evidence was based on cross—sectional designs and could not effectively observe the long-term cumulative impact of intergenerational support (21). From the few studies analyzing longitudinal data, a longitudinal data study from a clinical trial found that participants assessed at four time points within 12 months showed that emotional and practical support from family members could reduce the occurrence of psychological distress, thereby lowering the degree of depression (22).

Another limitation of the existing evidence is that the study population is a special group of the older adult, which leads to significant health disparities and makes it difficult to avoid endogeneity issues. There are significant health differences within the older adult group (such as chronic diseases, physical functional disorders, etc.), which may interact with mental health, making it difficult to completely exclude endogeneity problems. In other words, the research results may be affected by the health status within the older adult group, impacting the certainty of causal relationships. Although previous studies have revealed the association between family support and the mental health of the older adult, most of these studies are based on the general older adult population and lack sample analysis for specific health conditions (such as older adult patients with hypertension). This means that existing studies may not accurately reflect the relationship between family support and mental health in the special group of older adult patients with hypertension. Older adult patients with hypertension, due to their long-term illness, may face more psychological stress and emotional problems, such as anxiety and depression. Therefore, intergenerational family support may be of greater significance to their mental health.

This paper draws on the 2020 data from the China Health and Retirement Longitudinal Study (CHARLS) to fill the current evidence gap. The study aims to assess the changes in the mental health of older adult patients with hypertension who are in different categories of intergenerational family support.

## 2 Methods

### 2.1 Data

This study uses the 2020 data from the fifth wave of CHARLS, a longitudinal survey of mainland China’s population aged 45 and above. It collects multi—dimensional data on socio—economic and health status for aging research. Inspired by international aging surveys like the U. S. HRS, CHARLS ensures international data—collection standards. Its 2011–12 baseline survey covered 150 counties/districts, 450 villages/communities, 10,257 households, and 17,708 individuals, with follow-ups in 2013, 2015, 2018, and 2020 (23, 24).

**Figure 1:**
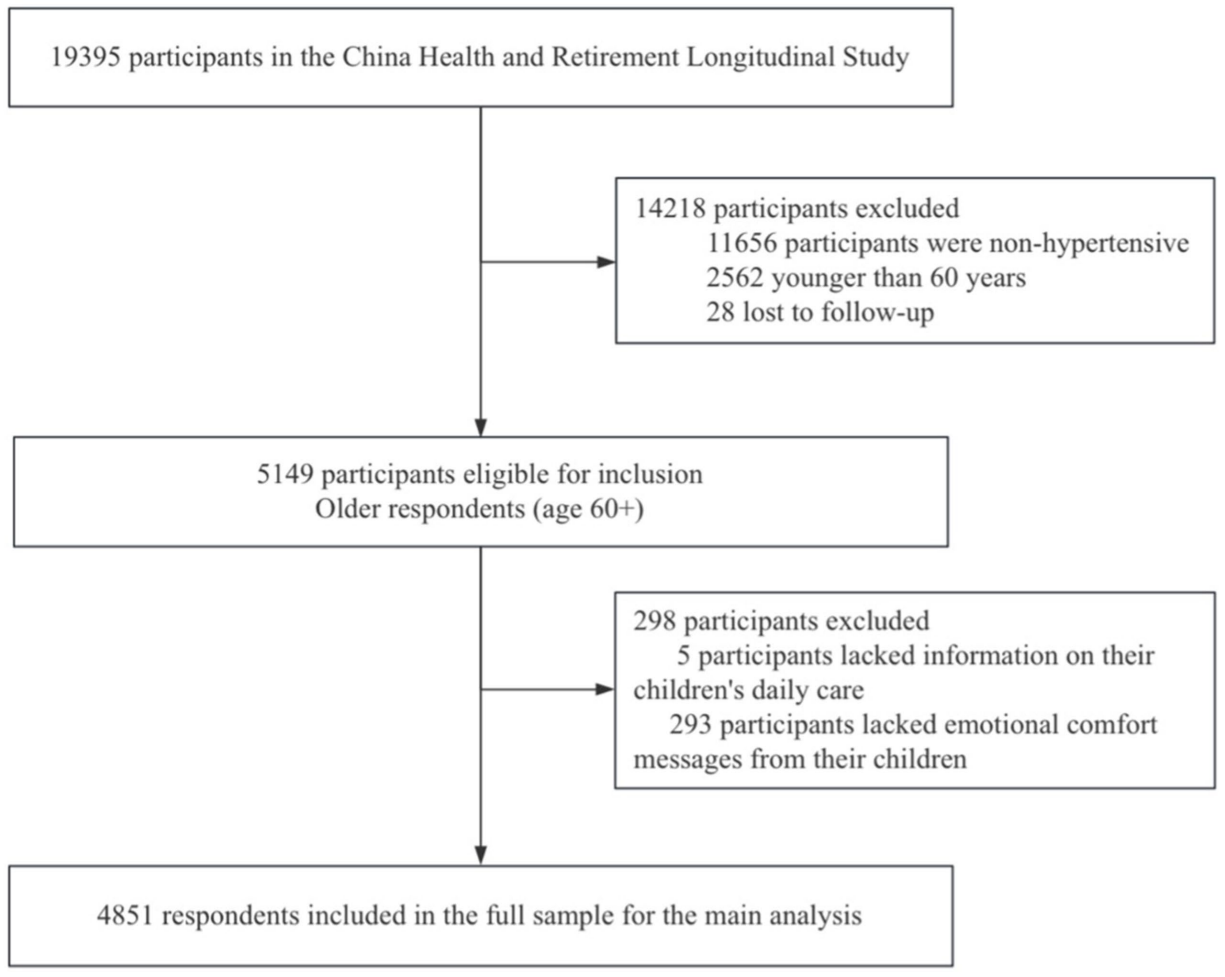
Study profile. *Note that some participants are included in both the lost-to-follow-up category and the incomplete data category.

The study subjects of this research are older adult patients with hypertension. Therefore, the inclusion criteria were patients who were diagnosed with hypertension in 2011, aged 60 years or above, and had at least one child. After excluding cases with missing values for key indicators, a total of 4,851 older adult patients with hypertension were included. For detailed information, please refer to Supplementary Table 1. The research overview is shown in Figure 1.

### 2.2 Measurement

#### 2.2.1 Independent variable

The core explanatory variable is intergenerational family support, consisting of caregiving, emotional, and financial support. Financial support is defined as “yes” (>0 yuan/year) or “no” (=0 yuan/year) based on the survey question. Caregiving support is determined by responses to questions about help with activities and future care. Emotional support is defined as “frequent contact” (≥ once a week) or “infrequent contact” (< once a week) by asking how often participants see or communicate with their children. For details, refer to Supplementary Table 2.

#### 2.2.2 Dependent variables

Depression is one of the most important indicators of mental health, and the academic community often uses it to measure the state of mental health. Individuals with a depressive tendency or depression are certain to have suboptimal mental health and may even have relatively severe psychological problems. Analyzing depressive tendencies can, to a certain extent, reflect the mental health status of the older adult, identify existing issues, and provide policy recommendations to improve the mental health of the older adult. This paper chooses depression as the measure of mental health and uses the Chinese version of the 10-item Center for Epidemiological Studies Depression Scale (CESD-10) to assess depressive symptoms. This scale has been used to measure depressive symptoms in the older adult and has been validated in Chinese older adult respondents (25). The total score ranges from 0 to 30, with higher scores indicating a higher level of depressive symptoms. A cutoff score of ≥ 10 is used to identify respondents with depressive symptoms (26).

#### 2.2.3 Control variables

The demographic and socioeconomic characteristics of older adult patients with hypertension, which are known potential factors affecting mental health, were included as control variables. These include personal characteristics such as gender, age, marital status, place of residence, and education level; socioeconomic characteristics such as social activities, internet usage, and pension insurance; and health—related behavioral characteristics such as living arrangements, smoking, drinking, activities of daily living (ADL), comorbidities and cognitive function (27).

## 3 Empirical model

Statistical analyses were conducted using Stata 17.0 software on the 2020 CHARLS data. Data were analyzed using the chi-square test for categorical variables and the independent t-test for continuous variables, with statistical significance defined as *p < 0.05 and **p < 0.01. The main analytical methods are as follows: Firstly, we constructed five models to analyze this association, with the depression score as the dependent variable and intergenerational family support as the independent variable. The first model is a univariate regression model. In models 2, 3, and 4, multiple linear regression models were established using the ordinary least squares (OLS) method to adjust for personal characteristics, socioeconomic characteristics, and health-related behavioral characteristics, respectively. Model 5 is also a multiple linear regression model that adjusts for all variables.

Ordinary Least Squares (OLS) Method as the Regression Model


(1) $${\text{score}}_{i}=\beta_{0}+\beta_{1}X_{1i}+\beta_{2}X_{2i}+\beta_{3}X_{3i}+\sum_{n}\;\beta_{n}K_{\mathrm{ni}}+\varepsilon_{i}$$

In Equation 1, score_i​_ represents the depression score of the i-th older adult hypertensive patient; X_1i_​ indicates whether the i-th older adult hypertensive patient receives financial support from the family; X_2i_​ indicates whether the i-th older adult hypertensive patient receives caregiving support from the family; X_3i​_ indicates whether the i-th older adult hypertensive patient receives emotional support from the family; K_ni​_ represents the set of control variables. β_1_​, β_2_​, and β_3_​ are the estimated coefficients corresponding to each variable, β_0_​ is the intercept term, and ε_i_​ is the random error term.

To address the issue of heteroskedasticity in the model, we used two methods for testing: the White test and the BP (Breusch–Pagan) test. In this study, we took the conclusion of the White test as the standard. With a *p*-value less than 0.05, it indicates the presence of heteroskedasticity. Therefore, we used the Robust standard error regression method in this study to address the issue of heteroskedasticity.

To avoid distortion of the results due to multicollinearity, we used Stata to test the Variance Inflation Factor (VIF). Generally, when the VIF value is greater than 10, it is considered that there is a serious problem of multicollinearity. The average value of the VIF calculated for this model was 1.02, with a maximum value of 1.58, which is less than 10. Therefore, the problem of multicollinearity can be ignored. The collinearity diagnosis is presented in Supplementary Table 3.

Secondly, we conducted sensitivity analysis using propensity score matching (PSM) to control for selection bias and verify the baseline model conclusions. Firstly, we estimated the propensity scores (representing the combined level of confounding factors) through a logistic regression model. Then, we grouped participants based on whether they received family support and performed 1:1 matching using the propensity scores (requiring exact equality or within a caliper width of 0.01) to pair those who received support with those who did not. This process constructed a pseudo—population with similar covariate distributions between the two groups. After matching, we conducted a balance test (calculating the standardized bias, with >10% indicating imbalance). Once balance was achieved, we estimated the counterfactual outcomes. For the untreated group, the counterfactual was the expected outcome if they had received support (estimated based on the matched treated group). Finally, we calculated the Average Treatment Effect on the Treated (ATT) by comparing the actual outcomes of the untreated group with their counterfactual outcomes, which represents the difference in outcomes between receiving and not receiving support.


(2) $$Y_{i}=Y0_{i}+(Y1_{i}-Y0_{i})T_{i}$$

(3) $$\mathrm{ATT}=E(Y1_{i}-Y0_{i}|T_{i}=1)=E(Y1_{i}|T_{i}=1)-E(Y0_{i}|T_{i}=1)$$

In Equation 2, when T_i​_ = 1, it represents the “receiving intergenerational support” group, while T_i​_ = 0 represents the “not receiving intergenerational support” group. In Equation 3, ATT represents the difference in depression scores for i-th patient between receiving intergenerational support (E(Y1_i_​∣T_i​_ = 1)) and not receiving intergenerational support (E(Y0_i_​∣T_i​_ = 1)). This difference signifies the net effect of receiving intergenerational support on the patients’ depression scores. However, E(Y0_i_​∣T_i​_ = 1) is unobservable. The PSM method can find an effective substitute for E(Y0_i​_∣T_i​_ = 1) in the “non-treatment group,” namely E(Y0_i_​∣T_i_​ = 0), to achieve a “counterfactual estimation.”

Finally, we performed a heterogeneity analysis via grouped regression with a multivariate linear regression model to examine variations in the impact of intergenerational family support on older adult hypertensive patients’ mental health across urban/rural areas, age groups, genders, and education levels.

## 4 Results

### 4.1 Descriptive analytics

Among the 4,851 participants, most received emotional comfort (79.49%) and financial support (93.59%), while fewer did not. Daily care receipt showed less variation. The average depression score was 8.89. Those not receiving daily care had higher scores (9.48 vs. 8.49, *p* < 0.01), as did those lacking emotional comfort (9.44 vs. 8.75, *p* < 0.01). Older adult patients without a spouse reported weaker emotional bonds with their children (32.86% vs. 24.43%, *p* < 0.01) but received more daily care (18.44% vs. 31.48%, *p* < 0.01). Rural residents had less emotional contact (85.03% vs. 75.03%, *p* < 0.01) but more financial support (77.62% vs. 22.38%, *p* < 0.01). Females received more daily care (57.28% vs. 42.72%, *p* < 0.01). Those with impaired daily activities depended more on daily care but had less emotional contact (41.61% vs. 34.00%, *p* < 0.01). Higher education correlated with more emotional support (illiteracy rate: 37.79% vs. 30.60%, *p* < 0.01), and those with emotional comfort had better cognitive function (11.53 ± 7.07 vs. 10.16 ± 6.83, *p* < 0.01) (Table 1).

**Table 1:** Descriptive statistics.

Variables	Emotional comfort		*p*	Economic support		*p*	Life care		*p*	Total (*n* = 4851)
	Infrequently (*n* = 995)	Frequent (*n* = 3856)		No (*n* = 311)	Yes (*n* = 4540)		No (*n* = 1979)	Yes (*n* = 2872)		
Living with a partner/spouse			0.07			0.17			0.09	
No	240(24.12)	828(21.47)		78(25.08)	990(21.81)		412(20.82)	656(22.84)		1068(22.02)
Yes	755(75.88)	3028(78.53)		233(74.92)	3550(78.19)		1567(79.18)	2216(77.16)		3783(77.98)
Age (years)	69.87 ± 7.09	69.80 ± 6.91	0.77	68.21 ± 6.66	69.92 ± 6.96	0.00**	68.90 ± 6.21	70.44 ± 7.35	0.00**	69.81 ± 6.95
Gender			0.95			0.43			0.00**	
Female	545(54.77)	2116(54.88)		164(52.73)	2497(55.00)		1016(51.34)	1645(57.28)		2661(54.85)
Male	450(45.23)	1740(45.12)		147(47.27)	2043(45.00)		963(48.66)	1227(42.72)		2190(45.15)
Spouse			0.00**			0.19			0.00**	
No	327(32.86)	942(24.43)		91(29.26)	1178(25.95)		365(18.44)	904(31.48)		1269(26.16)
Yes	668(67.14)	2914(75.57)		220(70.74)	3362(74.05)		1614(81.56)	1968(68.52)		3582(73.84)
Educational attainment			0.00**			0.00**			0.00**	
Illiterate	376(37.79)	1180(30.60)		106(34.08)	1450(31.94)		578(29.21)	978(34.05)		1556(32.08)
Primary school	209(21.01)	825(21.40)		44(14.15)	990(21.81)		434(21.93)	600(20.89)		1034(21.32)
Primary school not completed	245(24.62)	858(22.25)		67(21.54)	1036(22.82)		454(22.94)	649(22.60)		1103(22.74)
Middle school	114(11.46)	609(15.79)		50(16.08)	673(14.82)		327(16.52)	396(13.79)		723(14.9)
High school and above	51(5.13)	384(9.96)		44(14.15)	391(8.61)		186(9.40)	249(8.67)		435(8.97)
Smoking			0.06			0.31			0.23	
No	756(75.98)	3036(78.73)		236(75.88)	3556(78.33)		1530(77.31)	2262(78.76)		3792(78.17)
Yes	239(24.02)	820(21.27)		75(24.12)	984(21.67)		449(22.69)	610(21.24)		1059(21.83)
Drinking			0.87			0.68			0.07	
No	715(71.86)	2781(72.12)		221(71.06)	3275(72.14)		1399(70.69)	2097(73.02)		3496(72.07)
Yes	280(28.14)	1075(27.88)		90(28.94)	1265(27.86)		580(29.31)	775(26.98)		1355(27.93)
ADL			0.00**			0.12			0.00**	
Have difficulty with any of them	414(41.61)	1311(34.00)		98(31.51)	1627(35.84)		752(38.00)	973(33.88)		1725(35.56)
No difficulty	581(58.39)	2545(66.00)		213(68.49)	2913(64.16)		1227(62.00)	1899(66.12)		3126(64.44)
Pension insurance			0.62			0.72			0.47	
No	146(14.67)	510(13.23)		40(12.86)	616(13.57)		276(13.95)	380(13.23)		656(13.52)
Yes	849(85.33)	3346(86.77)		271(87.14)	3924(86.43)		1703(86.05)	2492(86.77)		4195(86.48)
Social events			0.00**			0.32			0.00**	
1 day a few months	148(14.87)	591(15.33)		43(13.83)	696(15.33)		276(13.95)	463(16.12)		739(15.23)
1–2 days a week	75(7.54)	369(9.57)		27(8.68)	417(9.19)		200(10.11)	244(8.50)		444(9.15)
Almost every day	148(14.87)	744(19.29)		48(15.43)	844(18.59)		335(16.93)	557(19.39)		892(18.39)
Never	624(62.71)	2152(55.81)		193(62.06)	2583(56.89)		1168(59.02)	1608(55.99)		2776(57.23)
Surf the web			0.00**			0.10			0.39	
No	886(89.05)	3030(78.58)		240(77.17)	3676(80.97)		1586(80.14)	2330(81.13)		3916(80.73)
Yes	109(10.95)	826(21.42)		71(22.83)	864(19.03)		393(19.86)	542(18.87)		935(19.27)
Area of residence			0.00**			0.00**			0.19	
Rural	846(85.03)	2893(75.03)		215(69.13)	3524(77.62)		1544(78.02)	2195(76.43)		3739(77.08)
Urban	149(14.97)	963(24.97)		96(30.87)	1016(22.38)		435(21.98)	677(23.57)		1112(22.92)
Comorbidities			0.03*			0.16			0.09	
No	145(23.81)	464(76.19)		47(7.72)	562(92.28)		229(37.60)	380(62.40)		609(12.55)
Yes	850(20.04)	3392(79.96)		264(6.22)	3978(93.78)		1750(41.25)	2492(58.75)		4242(87.45)
Cognitive function	10.16 ± 6.83	11.53 ± 7.07	0.00**	11.07 ± 7.14	11.26 ± 7.04	0.64	11.48 ± 6.74	11.09 ± 7.24	0.05	11.25 ± 7.04
Depression scores	9.44 ± 7.14	8.75 ± 6.62	0.00**	8.91 ± 6.95	8.89 ± 6.72	0.97	9.48 ± 6.92	8.49 ± 6.58	0.00**	8.89 ± 6.74

*p < 0.05, **p < 0.01. Continuous data are shown as mean ± standard deviations. Classified data is displayed as a number (%).

### 4.2 Baseline regressions

Using single—variable linear regression models, we found that compared to patients who did not receive intergenerational support, older adult patients who received daily care (*β* = −0.99, 95% CI [−1.37 to −0.60]) and those who received emotional connections (*β* = −0.68, 95% CI [−1.17 to −0.19]) had significantly lower depression scores. However, economic support was not found to affect depression scores (*β* = −0.01, 95% CI [−0.81 to 0.78]). In the four multivariable linear regression models, after adjusting for patient characteristics, socioeconomic factors, health behaviors, and all confounding factors, older adult patients who received daily care (β = −0.78, 95% CI [−1.15 to −0.42]) and emotional comfort (β = −0.56, 95% CI [−1.02 to −0.10]) still had significantly lower depression scores, while economic support remained insignificant (β = 0.01, 95% CI [−0.75 to 0.77]).

In summary, daily care and emotional comfort have significant protective effects on reducing depression scores. An increase of one unit of intergenerational support reduces the depression score by approximately 0.78 and 0.56 CES—D points, respectively. In contrast, the effect of economic support on depression scores is not significant (Table 2).

**Table 2:** Associations between intergenerational family support and depression scores across different models.

Variables	Model 1 (Univariate Model)		Model 2		Model 3		Model 4		Model 5	
	Coef.	95%CI	Coef.	95%CI	Coef.	95%CI	Coef.	95%CI	Coef.	95%CI
Life care	−0.99** (−5.04)	−1.37 ~ −0.60	−0.85** (−4.36)	−1.24 ~ −0.47	−1.00** (−5.04)	−1.39 ~ −0.61	−0.78** (−4.09)	−1.15 ~ −0.40	−0.78** (−4.17)	−1.15 ~ −0.42
Emotional comfort	−0.68** (−2.72)	−1.17 ~ −0.19	−0.49* (−2.02)	−0.98 ~ −0.01	−0.68** (−2.69)	−1.17 ~ −0.18	−0.87** (−3.63)	−1.34 ~ −0.40	−0.56* (−2.39)	−1.02 ~ −0.10
Economic support	−0.01 (−0.03)	−0.81 ~ 0.78	0.33 (0.82)	−0.46 ~ 1.11	0.15 (0.38)	−0.64 ~ 0.95	0.04 (0.10)	−0.73 ~ 0.81	0.01 (0.03)	−0.75 ~ 0.77
Male	−1.59** (−8.39)	−1.97 ~ −1.22	−1.72** (−8.06)	−2.14 ~ −1.30					−1.56** (−6.85)	−2.01 ~ −1.11
Age	−0.17** (−13.35)	−0.20 ~ −0.15	−0.19** (−13.39)	−0.22 ~ −0.16					−0.13** (−9.00)	−0.16 ~ −0.10
Spouses	−0.02 (−0.12)	−0.48 ~ 0.43	−0.71** (−2.88)	−1.20 ~ −0.23					−0.49 (−1.81)	−1.02 ~ 0.04
Urban	−1.01** (−4.77)	−1.42 ~ −0.59	−0.71** (−3.22)	−1.15 ~ −0.28					−0.87** (−4.03)	−1.29 ~ −0.45
Primary school	0.36 (1.25)	−0.20 ~ 0.94	0.61* (2.08)	0.04 ~ 1.19					0.71* (2.55)	0.16 ~ 1.26
Primary school not completed	1.05** (3.49)	0.46 ~ 1.65	0.82** (2.66)	0.22 ~ 1.43					1.52** (5.08)	0.93 ~ 2.10
Illiterate	0.37 (1.27)	−0.19 ~ 0.93	−0.04 (−0.12)	−0.67 ~ 0.59					1.56** (4.89)	0.94 ~ 2.19
High school and above	−0.73* (−2.12)	−1.40 ~ −0.05	−0.34 (−0.99)	−1.02 ~ 0.34					−0.53 (−1.64)	0.94 ~ 2.19
Socialize 1–2 days a week	−0.46 (−1.22)	−1.20 ~ 0.28			−0.49 (−1.31)	−1.23 ~ 0.25			−0.31 (−0.88)	−1.01 ~ 0.38
Socialize almost every day	−0.39 (−1.26)	−1.01 ~ 0.22			−0.37 (−1.19)	−0.99 ~ 0.24			−0.22 (−0.74)	−0.80 ~ 0.36
No social activities	−1.19** (−4.46)	−1.71 ~ −0.67			−1.24** (−4.63)	−1.77 ~ −0.72			−0.56* (−2.21)	−1.06 ~ −0.06
Surf the web	0.07 (0.33)	−0.35 ~ 0.49			−0.09 (−0.42)	−0.52 ~ 0.34			−0.58* (−2.43)	−1.04 ~ −0.11
Pension insurance	0.70* (2.49)	0.15 ~ 1.26			0.68* (2.42)	0.13 ~ 1.23			0.28 (1.06)	−0.24 ~ 0.79
Not living alone	−0.96** (−4.01)	−1.43 ~ −0.49					−1.08** (−4.77)	−1.52 ~ −0.63	−0.82** (−3.21)	−1.31 ~ −0.32
Smoking	−0.61** (−2.75)	−1.04 ~ −0.18					−0.64** (−2.89)	−1.07 ~ −0.21	0.04 (0.17)	−0.41 ~ 0.49
Drinking	−0.47* (−2.31)	−0.86 ~ −0.07					−0.81** (−4.00)	−1.20 ~ −0.41	−0.16 (−0.79)	−0.57 ~ 0.24
Normal ADL	−1.69** (−7.85)	−2.11 ~ −1.27					−2.11** (−10.07)	−2.52 ~ −1.70	−2.13** (−10.34)	−2.53 ~ −1.73
Comorbidities	2.41** (8.32)	1.84 ~ 2.98					1.69** (6.48)	1.18 ~ 2.20	1.70** (6.66)	1.20 ~ 2.20
Cognitive function	0.22** (17.01)	0.19 ~ 0.25					0.27** (20.15)	0.24 ~ 0.29	0.29** (19.51)	0.26 ~ 0.32
Observations	4,851		4,851		4,851		4,851		4,851	
R-squared	–		0.06		0.02		0.11		0.16	
F statistic	–		31.40^**^		8.23^**^		69.13**		48.74^**^	

Dependent Variable = Depression scores. *p < 0.05, **p < 0.01, *t*-value in parentheses. Model 1 is a univariate model for preliminary analysis. Model 2 is a multivariate model that includes gender, age, marital status, place of residence, and education level. Model 3 is a multivariate model that includes social activities, internet usage, and pension insurance. Model 4 is a multivariate model that includes living arrangements, smoking, alcohol consumption, activities of daily living (ADL), comorbidities and cognitive function. Model 5 includes all confounding variables. R^2^ reflects the model’s explanatory power, reporting the R^2^ value is sufficient, as the focus is often on whether X has a significant effect on Y rather than the magnitude of this effect.

### 4.3 Sensitive analysis

**Figure 2:**
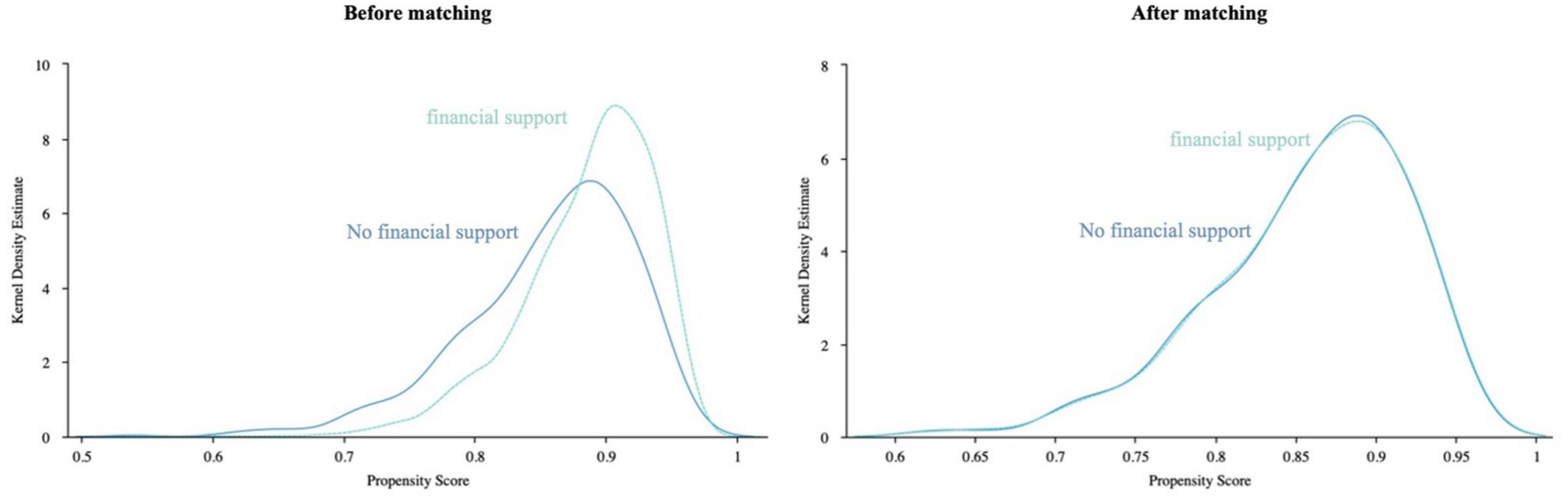
Kernel density function graph of the economic support group before and after matching.

**Figure 3:**
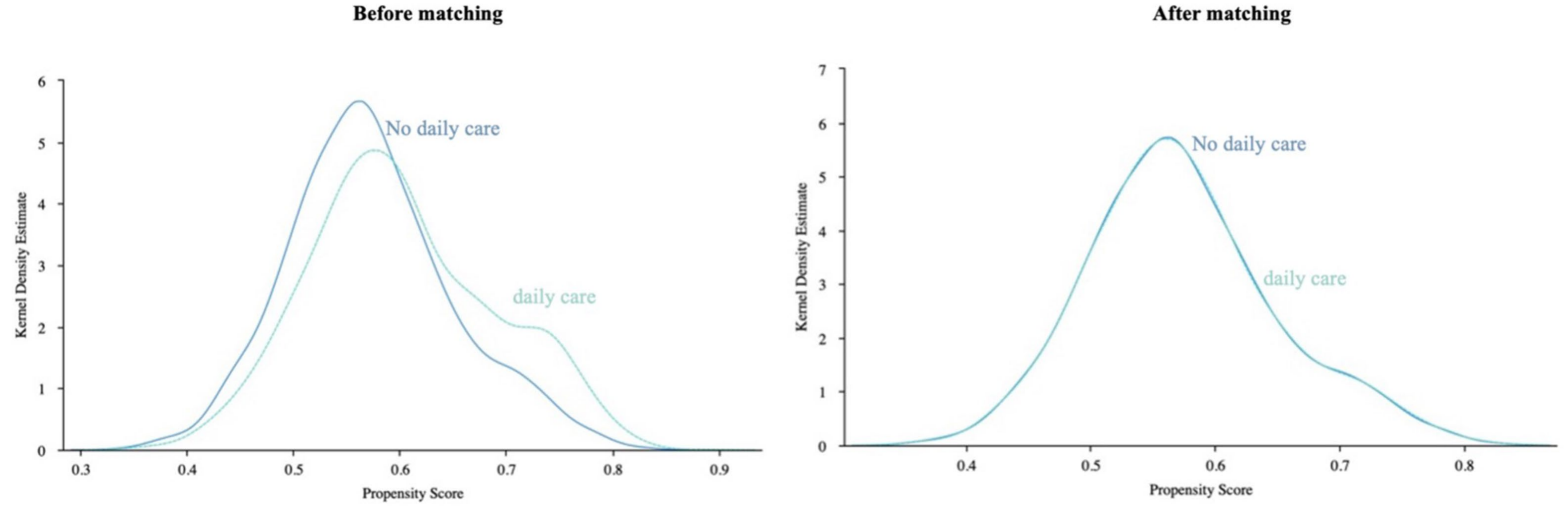
Kernel density function plot of support matching before and after for the daily care group.

**Figure 4:**
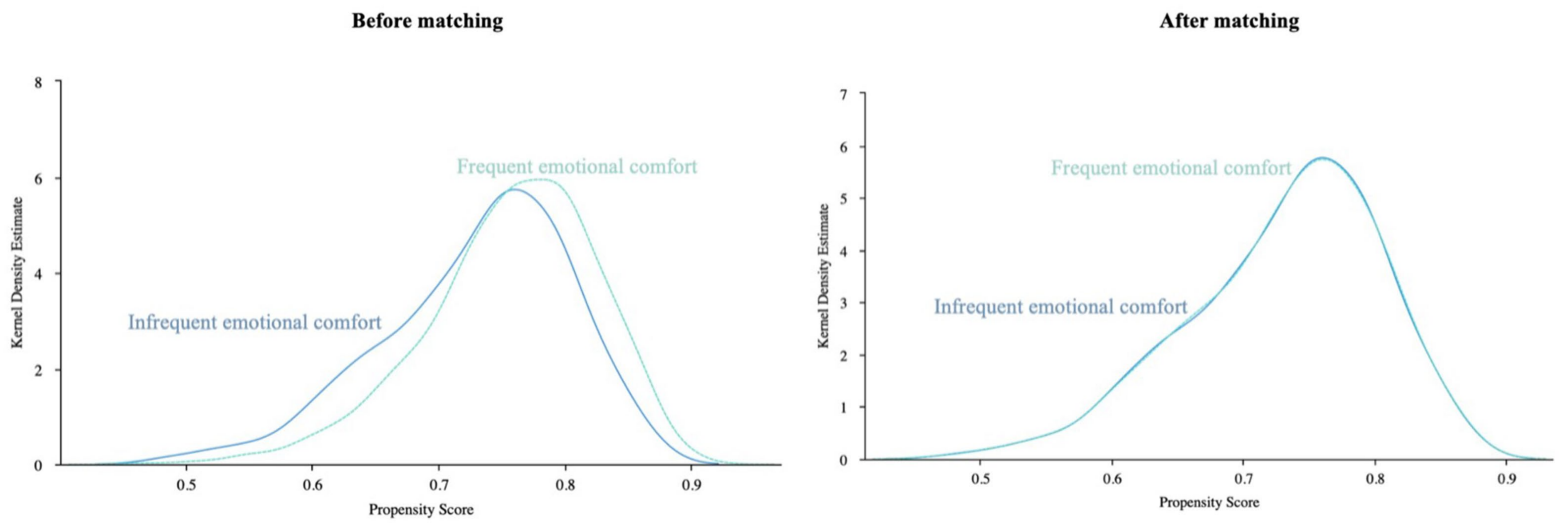
Kernel density function plot of the emotional comfort group before and after matching.

After PSM, we obtained 4,540 cases in the financial support group, 2,869 cases in the daily care group, and 3,853 cases in the emotional comfort group. Kernel density function plots before and after PSM matching (Figures 2–4). After matching, the kernel density distributions between the treatment and control groups tended to overlap, satisfying the common support test and indicating a good matching effect. Therefore, the matching process was proven to be effective. In Supplementary Table 3, the complete data of the PSM cohort are provided.

After counterfactual estimation using PSM, results showed that children’s emotional support reduced depression in older adult hypertensive patients by 78%, and daily caregiving alleviated it by 74%. Financial support had no significant effect on depression (Table 3).

**Table 3:** ATT effect analysis.

Caliper matching	Economic support			Life care			Emotional comfort		
	ATT effect	Std. error	*t*	ATT effect	Std. error	*t*	ATT effect	Std. error	*t*
	0.48	0.56	0.86	−0.74	0.22	−3.4^***^	−0.78	0.15	−5.11^***^

^***^p < 0.01.

The analysis shows that even after addressing selective bias in intergenerational support, emotional communication and daily care significantly boost patients’ psychological health, with emotional support having a greater impact than daily care. In contrast, economic support does not significantly affect the psychological health of older adult hypertensive patient.

### 4.4 Heterogeneity analysis

Our investigation explored the heterogeneous impact of community-based family intergenerational support on older adult hypertensive patients, categorizing the sample by age, gender, education, and residential area (28). Using grouped regression based on a multivariate linear regression model, we found significant differences in the effects of family support on mental health across these groups (Table 4).

**Table 4:** Heterogeneity analysis results.

Subgroups		Emotional comfort	Life care	Economic support
Age (years)	60–64	−1.23** (−2.62)	−0.76* (−2.03)	0.744 (1.09)
	65–69	−0.44 (−1.04)	−0.06 (−0.16)	0.16 (0.24)
	70–74	−0.89 (−1.74)	−0.05 (−0.11)	1.39 (1.41)
	75+	0.14 (0.27)	−2.26** (−5.30)	−1.05 (−1.12)
Gender	Male	−0.43 (−1.30)	−0.64* (−2.40)	0.03 (0.06)
	Female	−0.77* (−2.26)	−1.43** (−5.04)	0.27 (0.47)
Educational attainment	Illiterate	−0.18 (−0.41)	−1.35** (−3.46)	0.37 (0.49)
	Primary school not completed	−0.55 (−1.10)	−0.41 (−0.96)	−1.67 (−1.90)
	Primary school	−1.03* (−2.11)	−1.07** (−2.69)	0.44 (0.45)
	Middle school	−0.57 (−0.95)	−0.64 (−1.44)	1.76* (2.03)
	High school and above	−0.51 (−0.59)	−1.46** (−2.68)	0.07 (0.07)
Area of residence	Urban	−0.19 (−0.35)	−1.38** (−3.78)	−0.20 (−0.31)
	Rural	−0.59* (−2.19)	−0.82** (−3.58)	0.21 (0.43)

*p < 0.05, **p < 0.01, *t*-value in parentheses.

Specifically, family daily care significantly improved mental health for both genders, while emotional comfort mainly benefited women (*β* = −0.77, *p* < 0.05). Financial support had little impact on either gender’s mental health. Patients aged 75 and older showed a marked decrease in depression scores after receiving daily care (*β* = −2.26, *p* < 0.01), possibly due to increased dependence on such care. In contrast, for those aged 64 and younger, both daily care (*β* = −0.76, *p* < 0.05) and emotional comfort (*β* = −1.23, *p* < 0.01) significantly reduced depression scores. Overall, family intergenerational support mainly benefits mental health through daily care and emotional comfort.

Regarding education, daily care support significantly reduced depression scores among less educated patients. Urban and rural patients both benefited from daily care support, but emotional comfort had a more significant impact on rural patients (*β* = −0.59, *p* < 0.05).

## 5 Discussion

Our study reveals that intergenerational family support significantly impacts the psychological health of older adult hypertensive patients. Daily care and emotional comfort were found to improve mental health, with daily care reducing depression scores across multiple models and emotional support being particularly beneficial for women. Our results align with prior longitudinal studies on family support’s impact on older adult mental health (29). Daily care functions like blood pressure monitoring boost disease—management self—efficacy (30), and emotional comfort eases loneliness and hypertension—related stress, which fits the “stress—buffering model” (31). Emotional support exhibited unique benefits for female and rural patients, significantly alleviating depression in females (*β* = −0.77, *p* < 0.05) and rural patients (*β* = −0.59, *p* < 0.01). This may be because women are more open to expressing emotions (32), while rural areas have stronger traditional family ties where emotional support blends with daily care, forming a synergistic effect (33).

The results showed that financial support provided by children to older adult patients with hypertension was not significantly associated with their depression scores, but only slightly positively correlated (PSM—ATT effect: +0.48, *p* > 0.05). This finding challenges the traditional assumption that “financial assistance is necessarily beneficial” (34, 35). Reasons may be that financial support cannot resolve psychological stress caused by the disease, such as anxiety. Also, older adult Chinese often have stable pensions or medical insurance, so extra economic support brings few mental health benefits. Plus, in China’s filial piety culture, taking economic support from children might mean losing independence, lowering self—worth (36, 37). This is consistent with emerging evidence (38). So, economic support has limited and insignificant effects on depression in older adult hypertensive patients. This shows emotional support and daily care are very valuable for their mental health.

Poor mental health in older adult hypertensive patients can lead to blood pressure control issues, higher cardiovascular risks, worsened kidney damage, and cognitive decline. Depressive symptoms significantly reduce medication adherence in the older adult and increase stroke risk, particularly in women (39, 40). These patients also face a higher risk of clinical inertia (41). Insufficient family support may disadvantage older adult hypertensive patients in managing their condition.

To improve the psychological health of older adult individuals lacking intergenerational family support, various support pathways exist, such as community support, social participation programs, and mental health services and education (42, 43). However, targeted assistance for older adult hypertensive patients is insufficient. This is a significant issue because the needs of older adult hypertensive patients may differ from those of the general older adult population. Community resources may be inadequate to meet these specific needs, and community volunteers might lack the necessary professional medical and mental health support capabilities. Additionally, cognitive decline in older adult hypertensive patients can affect their understanding and application of mental health knowledge, and mental health education may not be sufficiently personalized to address the unique disease characteristics and psychological needs of hypertensive patients.

This study has several limitations. Using 2020 CHARLS cross—sectional data means we can only show the correlation between family intergenerational support and older adult hypertensive patients’ mental health, not causality. Reverse causality might exist, as those with poor mental health may depend more on family support. Unobserved confounders like children’s personality traits might also influence the relationship. The lack of longitudinal data restricts our analysis of dynamic and long-term effects. While PSM helps control selection bias, its effectiveness relies on matching variables and model assumptions. Unobserved confounders might still remain, and the smaller post-PSM sample size could weaken result robustness. Lastly, our grouped regression analysis might not fully capture individual heterogeneity in responses to intergenerational support across different education or socio-economic levels. Future research should explore these aspects further.

## 6 Recommendations

Based on the research findings, priority should be given to providing emotional and daily care support for older adult hypertensive patients, particularly for women, those aged 75 and above, and rural residents. Healthcare providers should be trained to identify depression in hypertensive patients using standardized tools and to advise families on non-material support strategies. Community services and family support systems should be strengthened to offer patients practical assistance and opportunities for emotional interaction.

## 7 Conclusion

The findings show that older adult hypertensive patients have better mental health when they receive emotional communication and daily care, while financial support has little effect. We confirmed the “asymmetric” effect of intergenerational support (daily care > emotional > financial) in hypertensive patients, offering a new angle for social support in chronic disease management. Based on these results and prior evidence, we should develop unique plans and strategies for older adult hypertensive patients.

## Data Availability

The datasets presented in this study can be found in online repositories. The names of the repository/repositories and accession number(s) can be found at: http://charls.pku.edu.cn.
