# Supplementary material for: Impacts of financial, caregiving, and emotional support on mental health: case of hypertensive patients in China
Source: Front Public Health. 2025 Jul 24;13:1601168. doi: 10.3389/fpubh.2025.1601168 (PMC12328140; doi:10.3389/fpubh.2025.1601168)
Supplement: Supplementary file 1 [file Table_1.docx]

**Supplementary Table 1. Construction of the definition of hypertensive patients.**

| Respondents | issue | Options and Answers | Definition of high blood pressure |
| --- | --- | --- | --- |
| People who have been diagnosed with hypertension at the last follow-up | Is your high blood pressure better than the time of your last visit, about the same or worse? | 1. Better  2. Worse  3. It's pretty much the same as the original  99. Did not have this condition on the last visit | Yes (other options)  No (option 99.) |
| New to the interviewee population and those who did not have hypertension at the previous follow-up | Has anyone ever told you you have high blood pressure? | 1. Yes 2. No | Same as before |

**Supplementary Table 2. Construction of the definition of core explanatory variables****.**

| variable | issue | Answers and option definitions | Whether or not to get a definition of support |
| --- | --- | --- | --- |
| Financial support | How much financial support did respondents and their spouses receive from their children in the past year when they were not living with their families? | How much money/things are given in total, and how much money/things are given on a regular basis | Yes (>0 RMB/year)  No (=0 RMB/year) |
| Care support | Who helped you in the above difficulties (dressing, bathing, eating, getting up, going to the toilet, housework, cooking, shopping, making phone calls, taking medicine, managing money, etc.)? | spouse  Parents, parents-in-law, father-in-law, mother-in-law  Children, daughters-in-law, grandchildren/grandchildren  Siblings and their spouses, children, and your spouse's siblings and their spouses, children  Other relatives  Employees (e.g. nannies)  Volunteers or members of voluntary agencies  Nursing home staff  Personnel of home care service institutions  Help from the community  Other personnel | Yes (Option 3)  No (other options) |
|  | If you need care for your daily life in the future, such as eating, dressing, having relatives or friends, will you be able to take care of you for a long time? Who is he/she you? |  |  |
| Emotional support | How often can you see your children when you don't live together? | 1. Almost every day  2. 2-3 times a week  3. Once a week  4. Once every half month  5. Once a month  6. Once every three months  7. Semi-annually  8. Once a year  9. Almost never  10. Miscellaneous | Frequent contact (> = once a week)  Infrequent contact (< once a week |
|  | When you and your children don't live together, how often do you contact them by phone, text message, WeChat, letter or email? |  |  |

| **Supplementary Table 3. Parallel hypothesis test for financial support.** | | | | | | | |
| --- | --- | --- | --- | --- | --- | --- | --- |
| Item | Status | Treatment group | Control group | Standardized Deviation (%) | Reduction in Standardized Deviation (%) | t | p Value |
| Residency arrangements | Before Matching | 1.78 | 1.75 | 7.73% | 10.01% | 1.29 | 0.20 |
|  | After Matching | 1.78 | 1.75 | 6.95% |  | 1.13 | 0.26 |
| Gender | Before Matching | 1.45 | 1.47 | -4.54% | -53.19% | -0.77 | 0.44 |
|  | After Matching | 1.45 | 1.48 | -6.96% |  | -1.16 | 0.25 |
| Marriage | Before Matching | 1.74 | 1.71 | 7.41% | 43.74% | 1.24 | 0.21 |
|  | After Matching | 1.74 | 1.72 | 4.17% |  | 0.69 | 0.49 |
| Educational attainment | Before Matching | 2.98 | 3.16 | -14.65% | 6.54% | -2.43 | 0.02 |
|  | After Matching | 2.98 | 3.15 | -13.69% |  | -2.21 | 0.03 |
| Smoking | Before Matching | 1.22 | 1.24 | -5.81% | -52.14% | -0.97 | 0.33 |
|  | After Matching | 1.22 | 1.25 | -8.84% |  | -1.44 | 0.15 |
| Drinking | Before Matching | 1.28 | 1.29 | -2.38% | -50.95% | -0.40 | 0.69 |
|  | After Matching | 1.28 | 1.29 | -3.60% |  | -0.59 | 0.55 |
| ADL | Before Matching | 1.64 | 1.68 | -9.16% | -15.67% | -1.58 | 0.11 |
|  | After Matching | 1.64 | 1.69 | -10.59% |  | -1.79 | 0.07 |
| Pension insurance | Before Matching | 1.86 | 1.87 | -2.08% | 51.04% | -0.36 | 0.72 |
|  | After Matching | 1.86 | 1.87 | -1.02% |  | -0.17 | 0.86 |
| Social events | Before Matching | 3.17 | 3.26 | -7.85% | 35.14% | -1.35 | 0.18 |
|  | After Matching | 3.17 | 3.23 | -5.09% |  | -0.85 | 0.40 |
| Internet | Before Matching | 1.19 | 1.23 | -9.34% | -5.63% | -1.55 | 0.12 |
|  | After Matching | 1.19 | 1.23 | -9.87% |  | -1.59 | 0.11 |
| Cognitive function | Before Matching | 11.26 | 11.07 | 2.72% | 52.85% | 0.46 | 0.65 |
|  | After Matching | 11.26 | 11.17 | 1.28% |  | 0.21 | 0.83 |
| Comorbidities | Before Matching | 1.88 | 1.85 | 7.94% | 6.95% | 1.31 | 0.19 |
|  | After Matching | 1.88 | 1.85 | 7.39% |  | 1.19 | 0.24 |

| **Supplementary Table 3. Parallel hypothesis test for care support** **(Continued).** | | | | | | | |
| --- | --- | --- | --- | --- | --- | --- | --- |
| Item | Status | Treatment group | Control group | Standardized Deviation (%) | Reduction in Standardized Deviation (%) | t | p Value |
| Residency arrangements | Before Matching | 1.77 | 1.79 | -4.90% | 80.11% | -1.68 | 0.09 |
|  | After Matching | 1.77 | 1.78 | -0.97% |  | -0.29 | 0.77 |
| Gender | Before Matching | 1.43 | 1.49 | -11.94% | 58.28% | -4.08 | 0.00 |
|  | After Matching | 1.43 | 1.45 | -4.98% |  | -1.46 | 0.14 |
| Marriage | Before Matching | 1.69 | 1.82 | -30.45% | 39.21% | -10.60 | 0.00 |
|  | After Matching | 1.69 | 1.77 | -18.51% |  | -5.54 | 0.00 |
| Educational attainment | Before Matching | 3.03 | 2.93 | 8.09% | 73.95% | 2.76 | 0.01 |
|  | After Matching | 3.03 | 3.00 | 2.11% |  | 0.62 | 0.54 |
| Smoking | Before Matching | 1.21 | 1.23 | -3.50% | 68.55% | -1.20 | 0.23 |
|  | After Matching | 1.21 | 1.22 | -1.10% |  | -0.32 | 0.75 |
| Drinking | Before Matching | 1.27 | 1.29 | -5.17% | 40.14% | -1.76 | 0.08 |
|  | After Matching | 1.27 | 1.28 | -3.09% |  | -0.90 | 0.37 |
| ADL | Before Matching | 1.66 | 1.62 | 8.59% | 14.59% | 2.93 | 0.00 |
|  | After Matching | 1.66 | 1.63 | 7.34% |  | 2.14 | 0.03 |
| Pension insurance | Before Matching | 1.87 | 1.86 | 2.09% | 63.15% | 0.71 | 0.48 |
|  | After Matching | 1.87 | 1.86 | 0.77% |  | 0.23 | 0.82 |
| Social events | Before Matching | 3.15 | 3.21 | -5.19% | 49.53% | -1.78 | 0.08 |
|  | After Matching | 3.15 | 3.18 | -2.62% |  | -0.77 | 0.44 |
| Internet | Before Matching | 1.19 | 1.20 | -2.50% | 60.02% | -0.85 | 0.39 |
|  | After Matching | 1.19 | 1.19 | -1.00% |  | -0.29 | 0.77 |
| Cognitive function | Before Matching | 11.09 | 11.48 | -5.64% | 41.57% | -1.94 | 0.05 |
|  | After Matching | 11.10 | 11.33 | -3.30% |  | -0.98 | 0.33 |
| Comorbidities | Before Matching | 1.87 | 1.88 | -5.04% | 34.28% | -1.73 | 0.08 |
|  | After Matching | 1.87 | 1.88 | -3.31% |  | -0.98 | 0.33 |
| Age | Before Matching | 70.44 | 68.90 | 22.61% | 37.74% | 7.86 | 0.00 |
|  | After Matching | 70.42 | 69.44 | 14.08% |  | 4.23 | 0.00 |
| Place of residence | Before Matching | 1.24 | 1.22 | 3.79% | 2.91% | 1.30 | 0.19 |
|  | After Matching | 1.24 | 1.22 | 3.68% |  | 1.09 | 0.28 |

| **Supplementary Table 3. Parallel hypothesis test for emotional support (Continued).** | | | | | | | |
| --- | --- | --- | --- | --- | --- | --- | --- |
| Item | Status | Treatment group | Control group | Standardized Deviation (%) | Reduction in Standardized Deviation (%) | t | p Value |
| Residency arrangements | Before Matching | 1.79 | 1.76 | 6.31% | -3.92% | 1.75 | 0.08 |
|  | After Matching | 1.79 | 1.76 | 6.56% |  | 1.72 | 0.09 |
| Gender | Before Matching | 1.45 | 1.45 | -0.20% | 94.86% | -0.06 | 0.95 |
|  | After Matching | 1.45 | 1.45 | -0.01% |  | -0.00 | 1.00 |
| Marriage | Before Matching | 1.76 | 1.67 | 18.73% | 20.83% | 5.13 | 0.00 |
|  | After Matching | 1.76 | 1.69 | 14.83% |  | 3.85 | 0.00 |
| Educational attainment | Before Matching | 2.98 | 3.04 | -5.56% | 3.99% | -1.62 | 0.11 |
|  | After Matching | 2.98 | 3.04 | -5.34% |  | -1.47 | 0.14 |
| Smoking | Before Matching | 1.21 | 1.24 | -6.58% | 5.01% | -1.83 | 0.07 |
|  | After Matching | 1.21 | 1.24 | -6.25% |  | -1.64 | 0.10 |
| Drinking | Before Matching | 1.28 | 1.28 | -0.58% | -83.74% | -0.16 | 0.87 |
|  | After Matching | 1.28 | 1.28 | -1.07% |  | -0.28 | 0.78 |
| ADL | Before Matching | 1.66 | 1.58 | 15.74% | 18.44% | 4.37 | 0.00 |
|  | After Matching | 1.66 | 1.60 | 12.83% |  | 3.37 | 0.00 |
| Pension insurance | Before Matching | 1.87 | 1.85 | 4.18% | -56.97% | 1.16 | 0.25 |
|  | After Matching | 1.87 | 1.84 | 6.56% |  | 1.71 | 0.09 |
| Social events | Before Matching | 3.16 | 3.25 | -8.84% | 33.25% | -2.49 | 0.01 |
|  | After Matching | 3.16 | 3.22 | -5.90% |  | -1.56 | 0.12 |
| Cognitive function | Before Matching | 11.53 | 10.16 | 19.77% | 13.35% | 5.62 | 0.00 |
|  | After Matching | 11.54 | 10.35 | 17.13% |  | 4.60 | 0.00 |
| Comorbidities | Before Matching | 1.88 | 1.85 | 7.48% | -21.72% | 2.06 | 0.04 |
|  | After Matching | 1.88 | 1.85 | 9.11% |  | 2.35 | 0.02 |
| Age | Before Matching | 69.80 | 69.87 | -1.01% | 10.01% | -0.28 | 0.78 |
|  | After Matching | 69.80 | 69.86 | -0.91% |  | -0.24 | 0.81 |

| **Supplementary Table 4. Collinearity diagnosis** | | |
| --- | --- | --- |
| Item | VIF | Tolerance |
| Residency Arrangements | 1.32 | 0.76 |
| Age | 1.36 | 0.74 |
| Gender | 1.56 | 0.64 |
| Marriage | 1.58 | 0.63 |
| Educational attainment | 1.12 | 0.90 |
| Smoking | 1.29 | 0.78 |
| Drinking | 1.23 | 0.81 |
| ADL | 1.14 | 0.88 |
| Depression scores | 1.18 | 0.85 |
| Emotional support | 1.05 | 0.95 |
| Financial support | 1.03 | 0.97 |
| Care support | 1.05 | 0.96 |
| Cognitive function | 1.48 | 0.68 |
| Pension insurance | 1.02 | 0.98 |
| Social events | 1.05 | 0.95 |
| Internet | 1.27 | 0.79 |
| Place of residence | 1.16 | 0.86 |
| Comorbidities | 1.05 | 0.95 |
